# Electronegative Low-density Lipoprotein Increases Coronary Artery Disease Risk in Uremia Patients on Maintenance Hemodialysis

**DOI:** 10.1097/MD.0000000000002265

**Published:** 2016-01-15

**Authors:** Chiz-Tzung Chang, Guei-Jane Wang, Chin-Chi Kuo, Ju-Yi Hsieh, An-Sean Lee, Chia-Ming Chang, Chun-Cheng Wang, Ming-Yi Shen, Chiu-Ching Huang, Tatsuya Sawamura, Chao-Yuh Yang, Nicole Stancel, Chu-Huang Chen

**Affiliations:** From the L5 Research Center, China Medical University (CMU) Hospital (C-TC, J-YH, A-SL, C-MC, M-YS, C-YY, C-HC); Division of Nephrology, CMU Hospital (C-TC, C-CK, C-CH); College of Medicine, CMU (C-TC, C-CK); Graduate Institute of Clinical Medical Science, CMU (G-JW, C-CW, M-YS); Department of Health and Nutrition Biotechnology, Asia University (G-JW), Taichung, Taiwan; Department of Epidemiology, Johns Hopkins Bloomberg School of Public Health, Baltimore, Maryland, USA (C-CK); Department of Medicine, Mackay Medical College, New Taipei, Taiwan (A-SL); Department of Physiology, Shinshu University School of Medicine, Matsumoto, Nagono, Japan (TS); Vascular and Medicinal Research, Texas Heart Institute, Houston, Texas, USA (NS, C-HC); Lipid Science and Aging Research Center, Kaohsiung Medical University (KMU) (C-HC); Center for Lipid Biosciences, KMU Hospital, KMU, Kaohsiung (C-HC); New York Heart Research Foundation, Mineola, New York, USA (C-HC); and Lipid and Glycoimmune Research Center, Changhua Christian Hospital, Changhua, Taiwan (C-HC).

## Abstract

Electronegative low-density lipoprotein (LDL) is a recognized factor in the pathogenesis of coronary artery disease (CAD) in the general population, but its role in the development of CAD in uremia patients is unknown. L5 is the most electronegative subfraction of LDL isolated from human plasma. In this study, we examined the distribution of L5 (L5%) and its association with CAD risk in uremia patients.

The LDL of 39 uremia patients on maintenance hemodialysis and 21 healthy controls was separated into 5 subfractions, L1–L5, with increasing electronegativity. We compared the distribution and composition of plasma L5 between uremia patients and controls, examined the association between plasma L5% and CAD risk in uremia patients, and studied the effects of L5 from uremia patients on endothelial function.

Compared to controls, uremia patients had significantly increased L5% (*P* < 0.001) and L5 that was rich in apolipoprotein C3 and triglycerides. L5% was significantly higher in uremia patients with CAD (n = 10) than in those without CAD (n = 29) (*P* < 0.05). Independent of other major CAD risk factors, the adjusted odds ratio for CAD was 1.88 per percent increase in plasma L5% (95% CI, 1.01–3.53), with a near-linear dose–response relationship. Compared with controls, uremia patients had decreased flow-mediated vascular dilatation. In ex vivo studies with preconstricted rat thoracic aortic rings, L5 from uremia patients inhibited acetylcholine-induced relaxation. In cultured human endothelial cells, L5 inhibited endothelial nitric oxide synthase activation and induced endothelial dysfunction.

Our findings suggest that elevated plasma L5% may induce endothelial dysfunction and play an important role in the increased risk of CAD in uremia patients.

## Abstract 2

Supplemental Digital Content is available in the text

## INTRODUCTION

The risk of coronary artery disease (CAD) is higher in uremia patients than in the general population.^1^ Although abnormal lipid metabolism is a known risk factor for cardiovascular disease in patients with metabolic syndrome,^2^ its role in the development of CAD in uremia patients remains controversial.^3^ In individuals with a high risk of CAD, atherosclerosis with endothelial dysfunction is frequently observed.^4^ The vascular endothelium is important for maintaining vascular tone and preventing atherosclerosis^5^; thus, uremia patients may have a higher risk of CAD due to a significant loss of vascular endothelial integrity.^6^ Hyperlipidemia, especially high serum levels of low-density lipoprotein (LDL) cholesterol (LDL-c), is a major risk factor for endothelial dysfunction and CAD in the general population.^7^ However, in uremia patients, serum LDL-c is usually within the normal range.^8^ Therefore, the use of LDL-c levels as a predictor of CAD risk may be neither as sensitive nor as specific for uremia patients as it is for the general population.

LDL is heterogeneous and can be classified by size, density, or electric charge.^9^ Uremia patients have higher serum levels of electronegative LDL than do healthy individuals.^10^ Electronegative LDL is more atherogenic and proinflammatory than its less electronegative counterparts,^11–13^ indicating that it has a potential role in promoting atherogenesis in the uremic milieu. However, whether electronegative LDL contributes to the increased risk of CAD in uremia patients has not been explored.

We have previously used fast-protein liquid chromatography with anion-exchange columns to separate LDL isolated from patients with homozygous familial hypercholesterolemia into 5 subfractions (L1–L5) with increasing electronegativity.^9^ L5, the most electronegative LDL subfraction, is naturally occurring—unlike oxLDL and cam-LDL—and is found at elevated levels in smokers and patients with acute myocardial infarction, diabetes, or hyperlipidemia.^14–17^ Recently, we showed that plasma levels of L5 were significantly elevated in patients with chronic kidney disease compared to healthy controls and that L5 disrupted calcium homeostasis, resulting in relaxation dysfunction of the heart in early chronic kidney disease.^18^ L5 is rich in apolipoprotein (Apo)C3^19^ and can induce endothelial apoptosis and the expression of lectin-like oxidized LDL receptor-1 (LOX-1), an atherogenic scavenger receptor present on the surface of endothelial cells, vascular smooth muscle cells, and macrophages.^19^ The upregulation of LOX-1 expression is associated with endothelial dysfunction and atherosclerosis.^20,21^ Furthermore, L5 uptake by LOX-1 into the endothelial cell cytosol inhibits endothelial nitric oxide synthase (eNOS) expression via the Akt signaling pathway. This, in turn, suppresses endothelial phospho-Akt and phospho-eNOS expression and results in decreased nitric oxide (NO) production, leading to endothelial cell apoptosis.^17,22,23^

In this study, we isolated L5 from uremia patients on chronic hemodialysis to determine whether the levels and composition of L5 were altered when compared with those of healthy individuals. In addition, we evaluated the effects of L5 isolated from uremia patients on endothelial function and examined the association between L5 and CAD in uremia patients.

## METHODS

### Patients

We designed a cross-sectional study to examine the association between L5% (percent L5 of total LDL) and CAD risk in uremia patients and to determine the effects of L5 from uremia patients on endothelial function. Our study included 39 adult patients with uremia who underwent maintenance hemodialysis (HD) for at least 6 months and 21 healthy volunteers with normal renal function. Smokers were excluded from the study. All study participants had complete records, including a medical history, a lipid profile, and an electrocardiogram. CAD was defined by the presence of at least 50% stenosis in at least one epicardial artery on coronary angiography.^24^ Study participants without CAD had no history of angina or regional ventricle wall motion abnormality on echocardiography. Informed consent was obtained from all participants. All procedures were approved by the China Medical University and Hospital Research Ethics Committee (reference number: DMR-100-IRB-224).

### LDL Isolation and Fractionation

Venous blood was collected after fasting before the initiation of HD therapy and was separated into plasma and cells immediately after retrieval. To avoid LDL oxidation in vitro, sodium azide (0.06% wt/vol), aprotonin (0.056 U/ml plasma), and EDTA (0.06% wt/vol) were added to each plasma sample. LDL was then isolated by using ultracentrifugation.^17^ Isolated LDL was desalted by dialyzing it against buffer A (0.02 M Tris–Cl [pH 8], 0.5 mM EDTA) 3 times for 24 hours at 4°C. Dialyzed LDL was then separated using by using fast protein liquid chromatography with a UnoQ12 column (Bio-Rad, Hercules, CA). Five LDL subfractions were eluted with multistep gradient buffer B (1 M NaCl in buffer A) according to electronegativity. L1 was the effluent collected between fractions 11 to 14 (18–28 minutes); L2, fractions 15 to 16 (28–32 minutes); L3, fractions 17 to 24 (32–48 minutes); L4, fractions 25 to 30 (48–60 minutes); and L5, fractions 31 to 40 (60–80 minutes). The absorbance of all subfractions was monitored at 280 nm.

### Agarose Gel Electrophoresis

To confirm different electric charges among the isolated LDL subfractions, 8 μg of each subfraction was loaded onto a 0.7% agarose gel (90 mM Tris, 80 mM borate, and 2 mM EDTA [pH 8.2]). Bovine serum albumin, which is negatively charged, was used as a reference. Gel electrophoresis was performed at 100 V for 2 hours, followed by Coomassie blue gel staining.^16^

### Analysis of LDL Subfractions

The protein concentration of L1 to L5 was determined by using the Lowry method.^25^ To determine the protein composition of each subfraction, 2 μg LDL protein was delipidated with ethyl acetate:ethanol (1:1), solubilized with 10% sodium dodecyl sulfate (SDS), and separated on a 4–12% bis–tris gel (Invitrogen, Grand Island, NY) at 135 V for 65 minutes at room temperature. Commercial human apolipoproteins were used as markers (Academy Bio-Medical, Houston, TX). Gels were then stained with Coomassie blue. Phospholipid, triglyceride, cholesterol, and cholesteryl ester masses were determined by using commercial kits (Wako Chemicals USA, Richmond, VA). All measurements were performed in duplicate.^17^ In the experimental portion of the study, we used the absolute concentration of L5 to estimate its cellular and physiologic effect. In the epidemiologic portion of the study, the absolute concentration of L5 was standardized by dividing it by each individual's total LDL concentration, yielding the percentage, or distribution, of L5 (L5%). We used L5% to examine the effect of L5 on endothelial function and its association with CAD.

### Aortic Ring Tension Assay

Thoracic aortas excised from 10 male Sprague–Dawley rats weighing 280 to 300 g were divided into four 3-mm-long rings.^26^ Before the tension experiment, rings from the same rat were placed in plates containing DMEM with L1 (100 μg/ml) or L5 (100 μg/ml) from HD patients or with vehicle for 6 hours. For some preparations, aortic rings were pretreated with LOX-1 neutralizing antibody, TS (30 μg/ml), for 60 minutes before L5 stimulation.

After treatment with LDL or vehicle, vascular tension was recorded by using a data acquisition system (PowerLab, ADInstruments Ltd, Denver, CO). Increasing concentrations of acetylcholine (10 nM–10 μM) were applied during the sustained phase (considered as 100%) of phenylephrine (0.3 μM)-induced contraction in the aortic rings. To estimate the contribution of endothelium-derived NO in the vasorelaxing effect of acetylcholine, the endothelium was disrupted in some preparations with a cotton swab, or preparations were pre-incubated with the NOS inhibitor l-NNA (100 μM) for 20 minutes before phenylephrine treatment.

### Western Blot Analysis

HAECs in EGM-2 medium were treated with L1 (50 μg/ml) or L5 (50 μg/ml) from HD patients or phosphate-buffered saline (PBS) and were cultured for 24 hours. Cellular proteins were extracted and subjected to SDS–polyacrylamide gel electrophoresis.^23^ Immunoblotting was performed with anti-LOX-1 (GeneTex, Inc., Irvine, CA), anti-Akt, anti-phospho-Akt, anti-eNOS, anti-phospho-eNOS (Ser1177), and anti-actin antibodies (Cell Signaling Technology, Danvers, MA). LOX-1 levels were normalized to those of β-actin, phospho-Akt levels were normalized to those of Akt, and phospho-eNOS levels were normalized to those of eNOS.

### Measurement of Flow-mediated Dilatation

Flow-mediated dilatation (FMD) was determined by inflating a sphygmomanometer cuff around the mid-forearm to 250 mm Hg for 5 minutes and then deflating the cuff. Brachial artery diameter was measured after cuff inflation and deflation from a B-mode ultrasound image (Logic e, GE Healthcare, Wauwatosa, WI). FMD was defined as the percent change of brachial artery diameter after cuff deflation.^27^

### Statistical Analysis

Univariate analysis of quantitative variables was performed by using a Student *t* test for data expressed as the mean ± SEM, or by using the Mann–Whitney *U* test for data expressed as the median value with the interquartile range (IQR, 25–75%). A Chi-square test was applied for categorical variables. For comparisons among multiple study groups, the analysis of variance (ANOVA) test was used. For the LDL subfraction composition study, differences between the 5 subfractions within either the HD group or the control group were analyzed by using the ANOVA test. Differences between corresponding LDL subfractions in the HD group and the control group were analyzed by using a *t* test.

We used multiple linear regression analysis to determine the association between FMD and L5%. A nonlinear regression curve was obtained by fitting a cubic polynomial to the data. We also performed semiparametric regression analysis to determine the functional relationship between L5% and FMD; the results of this analysis were consistent with the cubic polynomial fitting (data not shown). In addition, we used multiple logistic regression analysis to estimate the odds ratio of CAD in HD patients. Both linear and logistic regression models were adjusted for age, sex, diabetic status, calcium–phosphate product, and levels of high-sensitivity C-reactive protein (Hs-CRP).

All statistical analyses were performed in Stata/IC, version 12 (StataCorp, College Station, TX), and R, version 3.0.2 (R Foundation for Statistical Computing, Vienna, Austria). The 2-sided statistical significance level was set at α = 0.05.

## RESULTS

### Demographic Data and the Distribution of LDL Subfractions in Study Groups

The characteristics and serum profiles of uremia patients on maintenance hemodialysis (HD) (n = 39) and of healthy controls (n = 21) are shown in Table 1. In the HD group, 14 patients had diabetes mellitus and 10 patients had CAD, whereas patients in the control group had neither disease. The HD group had significantly higher median serum levels of Hs-CRP (*P* < 0.001), higher median triglyceride levels (*P* < 0.001), and lower median high-density lipoprotein cholesterol (HDL-c) levels (*P* < 0.001) than did the control group.

**TABLE 1:**
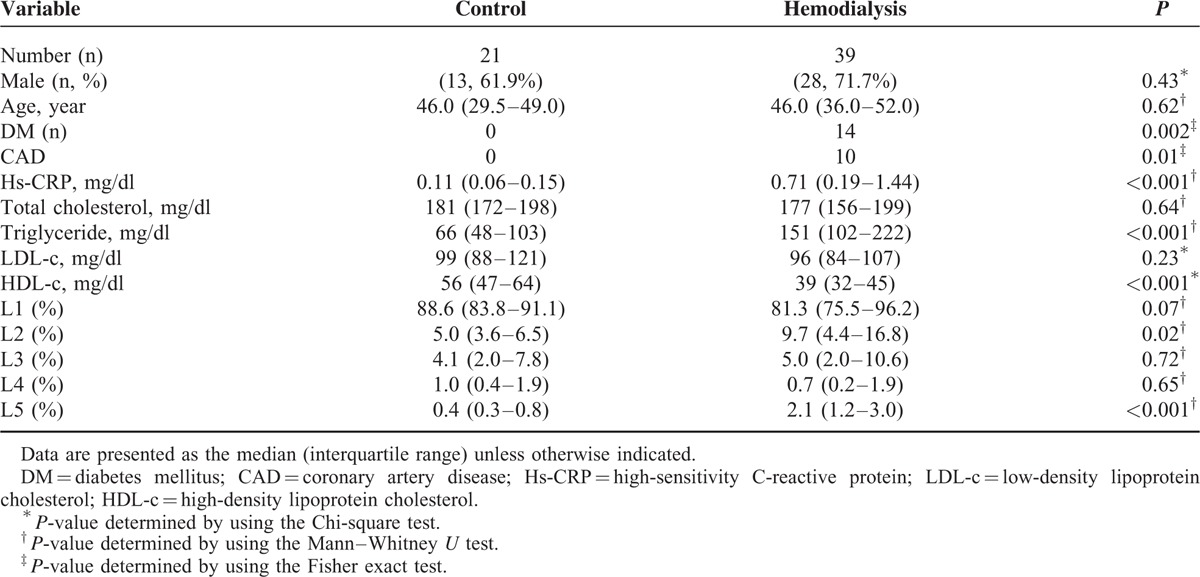
Characteristics and Serum Lipid Profiles of Uremia Patients on Hemodialysis and Controls

**FIGURE 1:**
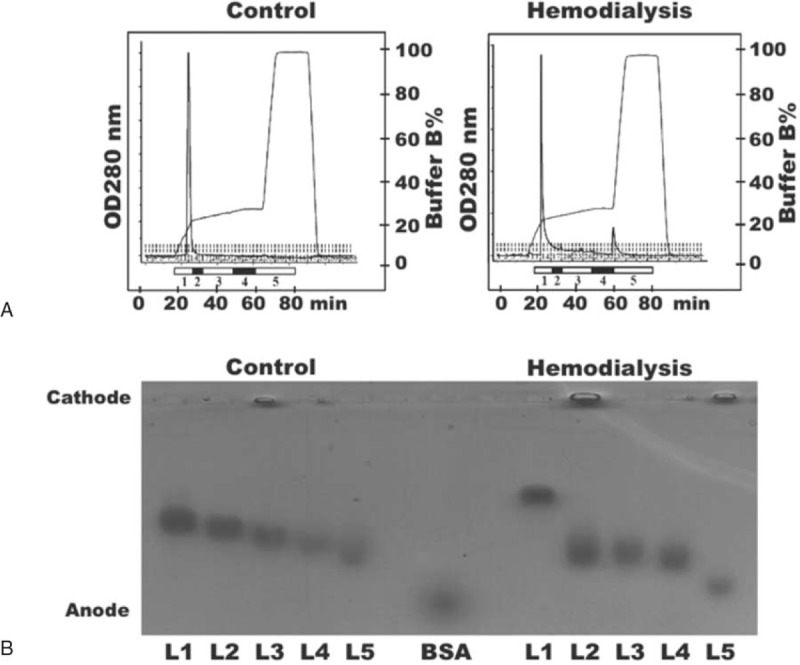
Distribution and electrophoretic mobility patterns of low-density lipoprotein (LDL) subfractions from controls and uremia patients on hemodialysis. (A) LDL from controls (left) and hemodialysis patients (right) was separated according to electronegativity by using fast-protein liquid chromatography with anion exchange columns. Five LDL subfractions, L1 to L5 (shown as 1–5), were collected at the indicated time points. (B) LDL subfractions were subjected to agarose gel electrophoresis at 100 V for 2 hours. L5 from both controls and hemodialysis patients migrated toward the anode faster than the other subfractions. Bovine serum albumin (BSA), which is negatively charged, was used as a reference.

LDL was separated into subfractions L1 to L5 according to electronegativity. Representative distribution patterns of LDL subfractions are shown in Figure 1A. L5 and L2 accounted for a significantly higher proportion of total LDL in the HD group than in the control group, but no significant difference was observed in the distribution of L1, L3, or L4 between groups (Table 1). The electromobility of LDL subfractions increased from L1 to L5 in the LDL of both HD patients and controls. However, L5 from HD patients migrated faster than did L5 from controls (Figure 1B). When we performed multiple regression analysis to identify factors associated with L5 distribution, we found that the serum triglyceride level was significantly associated with L5% in HD patients (Supplemental Figure 1).

### Distribution of L5 in HD Patients With or Without Diabetes Mellitus

Our previous study showed that L5% was higher in patients with diabetes mellitus than in healthy controls.^28^ However, we found that the L5% was comparable between HD patients with and without diabetes mellitus (Supplemental Table 1).

### Effects of L5% on CAD Risk in HD Patients

**FIGURE 2:**
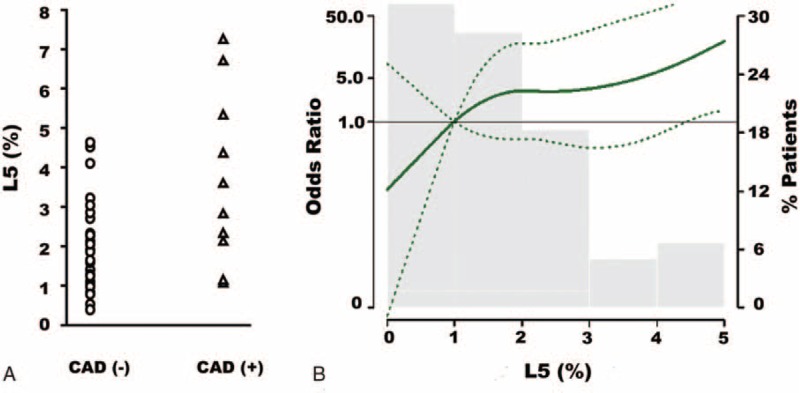
L5% and the risk of coronary artery disease (CAD) in uremia patients on hemodialysis (HD). (A) Scatter plots show the individual L5% values for HD patients without (−) or with (+) CAD. (B) The adjusted odds ratio of CAD per percent increase in L5% in HD patients was 1.86 (95% CI, 1.06–3.24). Variables adjusted: sex, age, diabetes mellitus, and calcium–phosphate product.

In HD patients with CAD (n = 10), L5% was higher than that in HD patients without CAD (n = 29) [3.1% (1.8–5.6) vs. 1.8% (1.1–2.5), *P* = 0.031] (Figure 2A). To determine whether L5% is an independent predictor of CAD, we used multiple logistic regression analysis to estimate the odds ratio of CAD in HD patients. The adjusted odds ratio per percent increase in L5% was 1.88 (95% CI, 1.01–3.53) in HD patients (Table 2). In dose–response analysis, the association of L5% with CAD was statistically significant, with no apparent departure from linearity (Figure 2B).

**TABLE 2:**
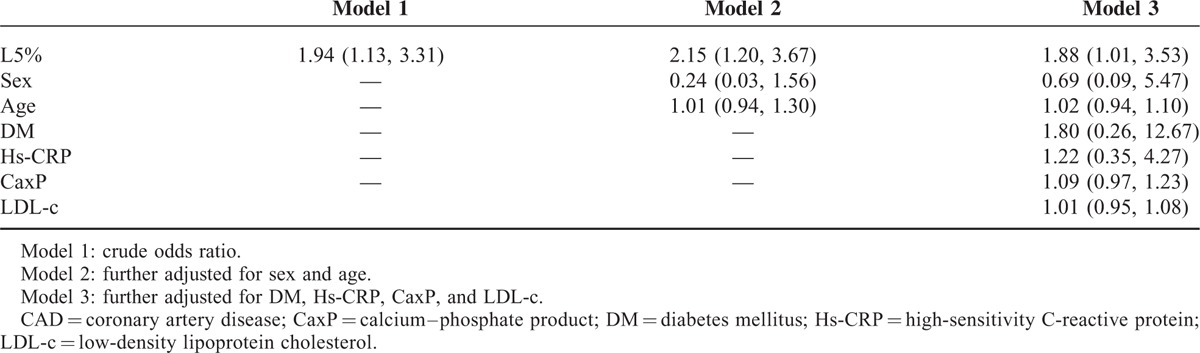
The Odds Ratios (95% Confidence Interval) of CAD by L5% in Hemodialysis Patients

### Analysis of FMD in HD Patients and Controls

**FIGURE 3:**
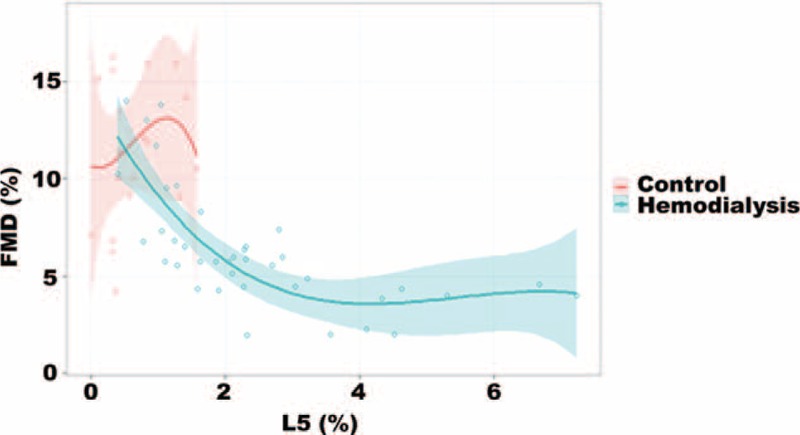
Flow-mediated dilatation (FMD) and its association with L5%. In uremia patients on hemodialysis, FMD decreased linearly with L5% to 4% and then leveled out. No association was observed between FMD and L5% in healthy controls.

FMD, which represents NO-dependent vascular dilatation, has been shown to be a predictor of arterial stiffness and a prognosticator of CAD.^29^ We found that FMD was significantly lower in HD patients than in healthy controls (5.7% [4.3–7.1] vs. 11.4% [9.0–15.2]; *P* < 0.001). To further define the functional relationship between L5% and FMD, we performed cubic polynomial approximation (Figure 3). Among HD patients, the FMD decreased linearly with L5% to 4% and then leveled out. No association was observed between FMD and L5% in healthy controls.

### Lipid and Protein Composition of L5 From HD Patients

In LDL from HD patients and controls, we examined and compared the masses of proteins and lipids for each LDL subfraction (L1 to L5). Pooled LDL subfractions were used for this purpose because the sample amount from a single individual was not adequate for analysis, especially for L4 and L5 subfractions. Therefore, we examined 6 pools of L1 to L5 (each pool contained 3–6 samples) from healthy controls and HD patients. We found no differences in the mean percentages (by mass) of protein, phospholipid, free cholesterol, or cholesteryl ester between any LDL subfractions. In addition, no differences were observed in these parameters between corresponding LDL subfractions from HD patients and controls (Table 3). However, the mean percentage of triglyceride in L5 was significantly higher in the HD group than in the control group (7.6% ± 0.5 vs. 5.2% ± 0.4) (*P* = 0.006), whereas the mean percentage of cholesteryl ester in L5 was significantly lower in the HD group than in the control group (21.5% ± 1.1 vs. 29.3% ± 1.8) (*P* = 0.005) In addition, the ratio of triglyceride/cholesteryl ester in L5 was significantly higher in the HD group than in the control group (0.363 ± 0.023 vs. 0.243 ± 0.024) (*P* = 0.023) (Table 3).

**TABLE 3:**
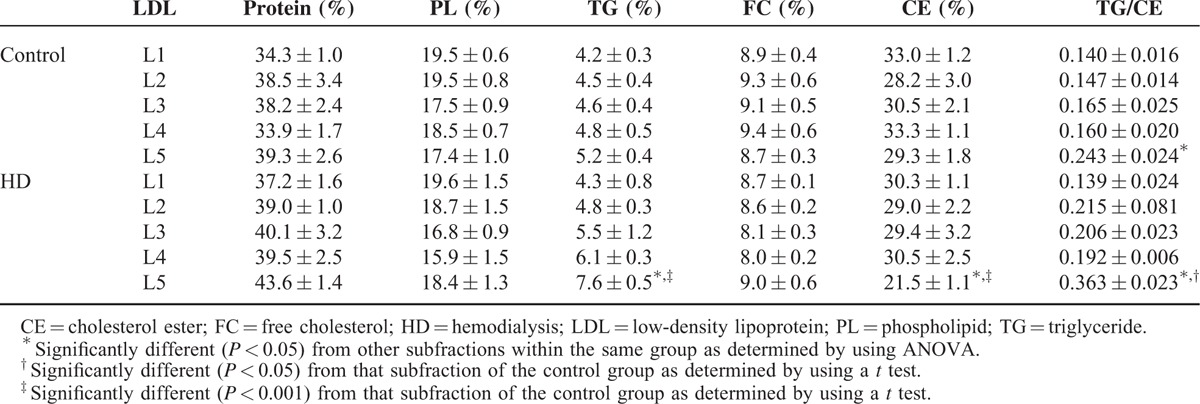
Percentage (by Mass) of Proteins and Lipids in LDL Subfractions L1–L5 From Hemodialysis Patients and Controls

**FIGURE 4:**
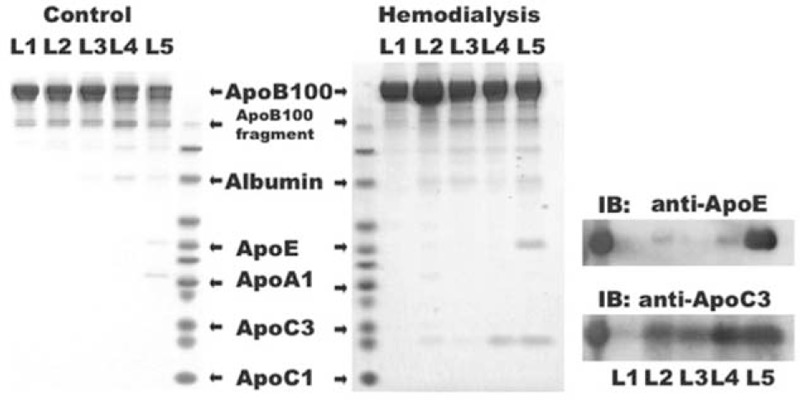
Apolipoprotein composition of low-density lipoprotein (LDL) subfractions in uremia patients on hemodialysis and healthy controls. Equal protein amounts of L1 to L5 were subjected to bis–tris gel electrophoresis, followed by Coomassie blue staining. ApoC3 and ApoE were more heavily stained in L5 of uremia patients on hemodialysis (middle panel) than in that of healthy controls (left panel). The results of Coomassie blue staining for uremia patients on hemodialysis were confirmed by immunoblot (IB) analysis of ApoE and ApoC3 in the same batch of LDL subfraction samples (right panel).

Using Coomassie blue staining, we examined the apolipoprotein composition of LDL subfractions (L1–L5) from healthy controls (Figure 4, left gel) and HD patients (Figure 4, middle gel). The amount of ApoE was higher in L5 of HD patients than in that of controls. In addition, the amount of ApoC3 was higher in all LDL subfractions from HD patients than in the corresponding subfractions from controls. Western blot analysis of ApoE and ApoC3 in LDL subfractions of HD patients showed that these apolipoproteins were mainly present in the more electronegative subfractions (Figure 4, right gel). Results of immunoblotting studies were consistent with the results from the Coomassie blue-stained gel.

### Less Prominent ApoB100 Fragmentation in L5 Than in oxLDL

Oxidized LDL has previously been shown to be a marker of cardiovascular disease.^30^ Serum levels of oxLDL are higher in patients with increased oxidative stress, such as diabetes, uremia, or CAD, than in normal controls.^31^ When we compared the fragmentation of ApoB100 in L5 and oxLDL, the results of SDS–polyacrylamide gel electrophoresis showed more prominent ApoB100 fragmentation in oxLDL than in L5 (Supplemental Figure 2).

### Effects of L5 From HD Patients on eNOS-dependent Vascular Relaxation

**FIGURE 5:**
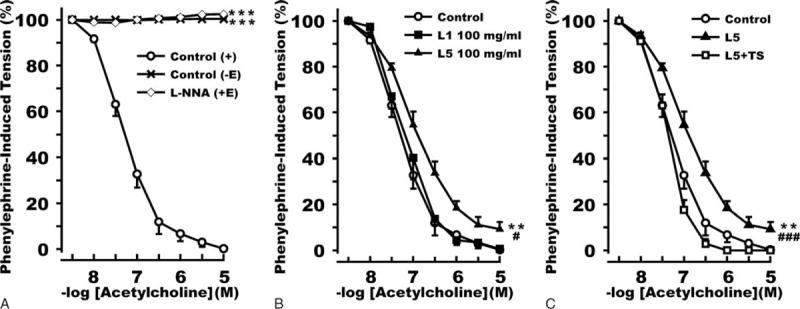
Effects of L5 from uremia patients on hemodialysis on acetylcholine-associated vascular relaxation. Aortic rings excised from rats were treated with L1 (100 μg/ml) or L5 (100 μg/ml) in DMEM medium for 6 hours, and 0.3 μM phenylephrine was added to induce vasoconstriction. Increasing concentrations of acetylcholine (10 nM–10 μM) were used to reverse the effect of phenylephrine. (A) Acetylcholine decreased phenylephrine-induced tension in a concentration-dependent manner in aortic rings with intact endothelium [control (+E), open circle], but the vasorelaxing effect of acetylcholine disappeared in aortic rings that were stripped of endothelium [control (−E), cross] and in endothelium-intact aortic rings costimulated with the nitric oxide synthase inhibitor l-NNA and acetylcholine [l-NNA (+E), open diamond]. ∗∗∗*P* < 0.001 versus control (+E). (B) Preincubation with L5 (100 g/ml) (black triangle) but not preincubation with L1 (100 g/ml) (black rectangle) for 6 hours blunted the vasorelaxing effect of acetylcholine in aortic rings. Aortic rings pre-incubated with DMEM for 6 hours were used as a negative control (open circle). ∗∗*P* < 0.01 versus control; ^#^*P* < 0.05 versus L1. (C) The addition of LOX-1 neutralizing antibody (TS) 1 hour before L5 treatment (L5 + TS, open rectangle) reversed the effect of L5 (black triangle) on aortic ring tension. DMEM treatment was used as a control (open circle). ∗∗*P* < 0.01 versus control; ^###^*P* < 0.001 versus L5 + TS. Each point represents the mean ± SEM values of 8 experiments. Analysis of serial levels was conducted by using nonparametric analysis of cumulative integrated area under the curve (AUC) measures for each specific condition.

To determine whether L5 from HD patients affects endothelial function, we examined acetylcholine-induced vascular relaxation by performing ex vivo studies in rat thoracic aortic rings. In aortic rings with intact endothelium, treatment with acetylcholine decreased aortic tension during the sustained phase of phenylephrine-induced contraction in a concentration-dependent manner (Figure 5A). In contrast, the denuding of the aortic endothelium completely attenuated acetylcholine's vasorelaxing effect (Figure 5A). We observed similar results in aortic rings with intact endothelium that were cotreated with NOS inhibitor N^ω^-nitro-l-arginine (l-NNA) (Figure 5A). In aortic rings pretreated for 6 hours with L5 (100 μg/ml) but not in those pretreated with L1 (100 μg/ml), the effect of acetylcholine on aortic tension was weakened (Figure 5B). When aortic rings were pretreated with LOX-1 neutralizing antibody (TS) for 1 hour before L5 treatment, this effect of L5 was attenuated (Figure 5C). These results indicate that L5 from HD patients impairs endothelium-dependent vascular relaxation via LOX-1 through an eNOS-dependent mechanism.

### Effects of L5 From HD Patients on LOX-1 and eNOS Expression

**FIGURE 6:**
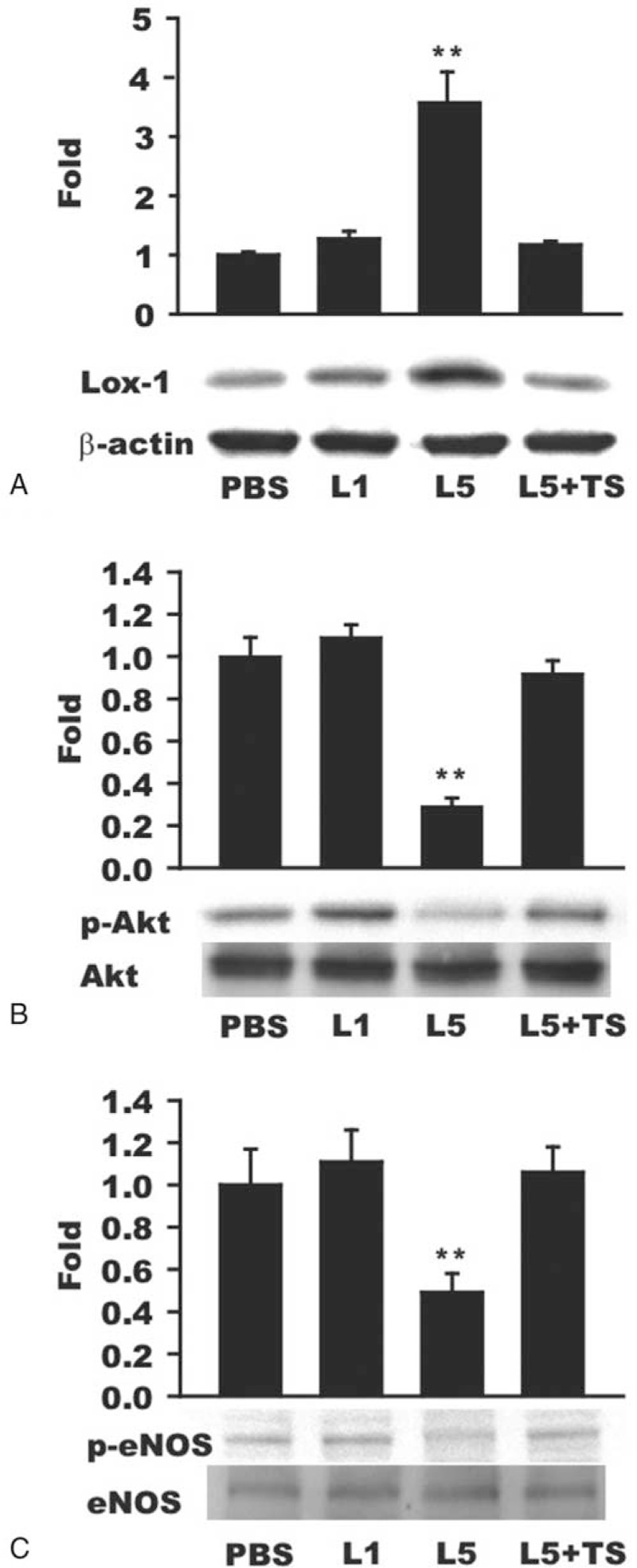
The inhibitory effects of L5 from uremia patients on hemodialysis on eNOS phosphorylation via the LOX-1/Akt pathway. Human aortic endothelial cells (HAECs) were treated with L1 (50 μg/ml) or L5 (50 μg/ml) from uremia patients on hemodialysis in the presence or absence of LOX-1 neutralizing antibody (TS) (30 μg/ml) for 24 hours. Phosphate-buffered saline (PBS)-treated HAECs served as a negative control. Western blot analysis of (A) LOX-1, (B), phospho-Akt (p-Akt), and (C) phospho-eNOS (p-eNOS) proteins is shown. In HAECs treated with L5 but not in cells treated with L1, LOX-1 expression was upregulated, whereas p-Akt and p-eNOS expression was inhibited. These effects of L5 were not observed in samples coincubated with TS. For each protein, results shown are representative of 4 independent experiments. ∗∗*P* < 0.01 versus PBS-treated control.

To further characterize the eNOS-dependent mechanism by which L5 from HD patients causes endothelial dysfunction, we compared the effects of L5 and L1 from HD patients on the expression of LOX-1, phospho-Akt, and phospho-eNOS in cultured HAECs. LOX-1 expression was significantly higher in L5-treated cells than in L1- or PBS-treated cells (*P* = 0.009; Figure 6A). When HAECs were treated with both L5 and LOX-1 neutralizing antibody (L5 + TS), L5-induced LOX-1 expression was attenuated (Figure 6A), indicating that the effect of L5 on LOX-1 expression was inhibited by LOX-1 neutralizing antibody. In addition, phospho-Akt and phospho-eNOS expression was significantly lower in L5-treated HAECs than in PBS-treated HAECs (*P* = 0.008 and *P* = 0.034, respectively; Figure 6B and C), whereas the expression of phospho-Akt and phospho-eNOS was not significantly different between HAECs treated with PBS and those treated with L1 or L5 + TS (Figure 6B and C). Given that the phosphorylation of eNOS occurs downstream of Akt phosphorylation, these findings suggest that L5 decreases eNOS phosphorylation through a signaling pathway involving LOX-1 and Akt.

## DISCUSSION

In this study, we showed that plasma L5% was significantly higher in LDL isolated from HD patients than in that from healthy controls with normal renal function. In addition, we found that the percentages of triglyceride, ApoE, and ApoC3 were higher in L5 from HD patients than in L5 from controls. Results from ex vivo analyses showed that L5 from HD patients impaired endothelium-dependent vasorelaxation in rat thoracic aortic rings. In vitro studies further demonstrated that L5 may cause endothelial dysfunction by suppressing the phosphorylation of eNOS through LOX-1 and Akt signaling pathways. In the clinical portion of this study, L5% was associated with increased arterial stiffness and an increased risk of CAD. Together, these findings indicate that lipid modification in the uremic milieu, particularly with respect to electronegative L5 LDL, plays an important role in the development of atherosclerosis and CAD in HD patients.

It was previously shown that uremia patients have higher serum levels of electronegative LDL than do healthy individuals, as demonstrated with an enzyme-linked immunosorbent assay.^10^ Consistent with these findings, we showed that L5% is elevated in uremia patients compared to healthy controls. One possible cause of elevated plasma L5 levels in HD patients may arise from the microinflammatory state that occurs in uremia. Levels of plasma CRP are elevated in 30% to 50% of uremia patients,^32^ which may result from uremic toxins, a bioincompatible hemodialyzer, acidosis, or other causes.^32^ Recently, we have reported that L5 induces the expression of CRP in serum,^14^ but the cause–effect relationship between L5 and CRP requires longitudinal investigation.

ApoC3-rich LDL and triglyceride-rich LDL are known risk factors for cardiovascular disease.^33,34^ Our findings indicated that L5 from HD patients is both ApoC3- and triglyceride-rich. In L5 from HD patients, the percentage of triglyceride was higher than that in any other LDL subfraction from either uremia patients or healthy controls. Moreover, the L5% in HD patients positively correlated with serum triglyceride concentration. High serum triglyceride levels can results from frequent heparin use and elevated parathyroid hormone.^35^ In uremia patients, hypertriglyceridemia can also result from a relative increase in plasma ApoC3 (an inhibitor of lipoprotein lipase) and a relative decrease in plasma ApoC2 (an activator of lipoprotein lipase).^8^ Our immunoblotting results showed that ApoC3 levels were higher in L5 from HD patients than in any other LDL subfraction. ApoC3-containing LDL inhibits the catabolism of apolipoprotein B lipoproteins and enhances the adherence of mononuclear cells to endothelial cells. Therefore, LDL rich in ApoC3 is proatherogenic.^36,37^ Clinical studies have shown that ApoC3-rich LDL can predict coronary events in the general population or in patients with diabetes mellitus.^34,38^ Interestingly, our previous studies have shown that L5 in patients with high CAD risk, such as those with diabetes mellitus or hyperlipidemia, also has a high triglyceride content.^17^ Despite the apparent correlation between serum triglyceride and L5%, the clinical significance of this correlation remains undetermined, and the clinical benefits of treating hypertriglyceridemia in the general population has not been unanimously validated.^39^

In addition to ApoC3, our results showed that ApoE was present at higher amounts in L5 from HD patients than in L5 from controls. ApoE is a ligand for several hepatic receptors such as the LDL receptor and LDL receptor-related protein, and is catabolized by these receptors.^40^ In uremia patients, LDL receptor function is impaired^41^; thus, L5 in these patients has a high ApoE content. Because the basal isoelectric points of ApoC3 and ApoE are 5.23 and 5.65, respectively, both of these apolipoproteins carry a negative charge under physiologic conditions.^42^ The enrichment of negatively charged ApoC3 and ApoE in L5 from HD patients may partly explain its increased electromobility when compared with L1–L4.

In our study, we also found that L5 from HD patients had a higher ratio of triglyceride/cholesteryl ester than did L5 from healthy controls. LDL with a high ratio of triglyceride/cholesteryl ester is susceptible to oxidation.^43^ Therefore, elevated L5 levels may be associated with high levels of oxidative stress seen in these individuals. However, as we previously reported,^17^ the degree of oxidation in L5 is much lower than that in oxLDL. Artificially oxidized oxLDL can be distinguished from naturally occurring L5 LDL by its higher degree of fragmented ApoB100, as seen in our SDS gel electrophoresis analysis.

Reduced vascular NO production can impair vasodilatation and lead to increased arterial stiffness and rigidity.^44^ In our aortic ring tension experiments, we found that endothelial dysfunction could be induced when inhibiting eNOS activity. Endothelial NOS is the main enzyme responsible for the production of NO, which counteracts the vasoconstricting effect of adrenergic hormone and neurological signals.^45^ Decreased cellular NO production may lead to endothelial cell apoptosis.^46^ Thus, the results from our in vitro experiments suggest that L5 may cause endothelial dysfunction by suppressing the phosphorylation of eNOS, which may in turn reduce NO production in patients and animals with uremia.^47,48^

Our previous studies have shown that L5 from patients with diabetes mellitus or hyperlipidemia inhibits endothelial cell eNOS phosphorylation via LOX-1.^49^ In the present study, we also showed that L5 but not L1 from HD patients suppressed eNOS activation through a LOX-1 and Akt signaling pathway. Like inflammation, treatment with L5 can also enhance the expression of LOX-1 in endothelial cells.^50^ LOX-1 is regulated by a cyclic adenosine monophosphate signaling pathway and is involved in the regulation of cellular apoptosis.^23^ LOX-1 activation may increase the formation of cellular tumor necrosis factor-alpha (TNF-α), which acts through its receptor to induce endothelial cell apoptosis.^51^ In addition, LOX-1 activation has been shown to inhibit Akt phosphorylation, which in turn decreases production of the antiapoptotic protein Bcl-2 and increases production of proapoptotic proteins such as Bad and Bax to promote endothelial cell apoptosis.^23^ Moreover, our previous studies have shown that L5 induces endothelial cell apoptosis via the activation of LOX-1,^16,49^ which we also confirmed in a separate set of experiments using L5 from HD patients (unpublished data). Although L5 composes only a small proportion of LDL, the endothelial injury triggered by L5 may be augmented through its signal transduction pathways. Consistent with this notion, we have previously reported that L5 may activate platelets and release proinflammatory cytokines, leading to endothelial damage.^14^

Our study showed that L5 reduced endothelial cell NO production, which leads to impaired vascular relaxation.^52^ The results of these experiments are consistent with our clinical data in HD patients showing that plasma L5% was negatively associated with FMD, a specific marker of NO-related endothelial function.^53^ This translational finding provides a new insight into the role of lipids in the underlying mechanism of atherosclerosis in patients with chronic kidney disease.

## LIMITATIONS

In our study, we performed high-quality outcome surveillance for CAD and conducted rigorous laboratory methods for quantifying LDL subfractions, which required at least 20 ml of blood from each HD patient. However, our study has several limitations. First, the epidemiologic finding showing the causal influence of L5% on the risk of CAD is confined by the cross-sectional design of our study. Therefore, our translational research findings, combined with our basic research findings, limit susceptibility toward reverse causation bias. Second, our patient sample size was relatively small; therefore, our results may be biased by inflated effects, and the possibility of chance finding cannot be ruled out. However, we present a clear dose–response relationship with concordant mechanistic evidence to corroborate the unique role of L5 in the development of cardiovascular disease in patients with HD. Finally, decreased renal function is a well-known risk factor of CAD^54^; however, we were not able to analyze the interaction of L5% and renal function on CAD because only HD patients and healthy volunteers were included in this study. Other limitations of our study include the possibility of residual confounding factors in the causal pathway, such as CRP. Consistent findings from our serial sensitivity analyses support the robustness of our translational findings.

## CONCLUSIONS

In conclusion, we showed that L5 from patients with uremia directly inhibits eNOS activation in vitro and impairs NO-dependent arterial relaxation in rat aortic rings. In the uremic milieu, the level and composition of human L5 are altered and are associated with endothelial dysfunction and increased risk of CAD, independent of diabetes, calcium–phosphate product, and Hs-CRP. Thus, our translational study highlights a novel mechanism of lipid modification in uremia that leads to atherosclerosis. To evaluate the therapeutic potential of targeting L5 in cardiovascular disease prevention, large prospective studies with long-term follow-up are needed.

## OTHER INFORMATION

### Statement of Nonduplication

The authors state that this manuscript is a unique submission that is not being considered for publication by any other source in any medium. Further, this manuscript has not been published, in part or in full, in any form.

## Supplementary Material

Supplemental Digital Content
