# Supplementary material for: Electronegative Low-density Lipoprotein Increases Coronary Artery Disease Risk in Uremia Patients on Maintenance Hemodialysis
Source: Medicine (Baltimore). 2016 Jan 15;95(2):e2265. doi: 10.1097/MD.0000000000002265 (PMC4718229; doi:10.1097/MD.0000000000002265)
Supplement: Supplemental Digital Content [file medi-95-e2265-s001.pdf]

Supplemental Digital Content

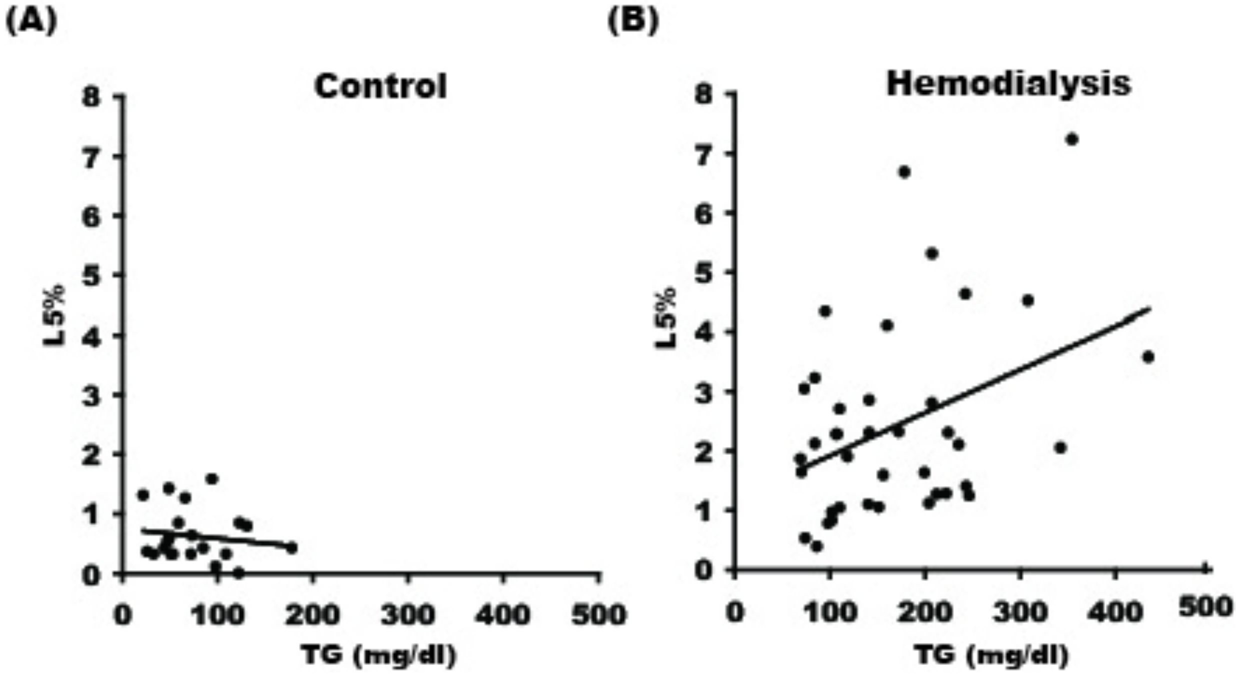

Supplemental Figure 1
Positive correlation of L5% with serum triglyceride levels in uremia patients on hemodialysis. Scatter plots show that L5% significantly correlated with serum triglyceride (TG) levels in uremia patients on hemodialysis (*p*=0.035) but not in healthy controls (*p*=0.127), after adjusting for age, sex, diabetes mellitus, and high-sensitivity C-reactive protein.

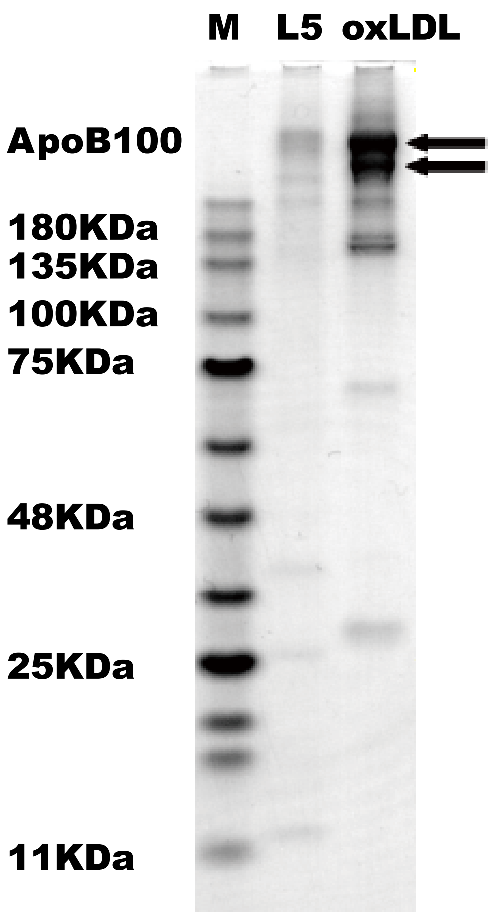

Supplemental Figure 2
Fragmentation of apolipoprotein B100. Equal amounts (7 μg) of L5 from uremia patients on hemodialysis and oxLDL were subject to sodium dodecyl sulfate (SDS) gel electrophoresis. More prominent apolipoprotein B100 fragmentation was observed in oxLDL than in L5 (arrows). M represents the marker.

Supplemental Table 1. Demographic and plasma parameters of healthy controls, uremia patients on hemodialysis without diabetes, and uremia patients on hemodialysis with diabetes.

**Variables**	**(1) Healthy controls N=21**	**(2) Hemodialysis without diabetes N=25**	**(3) Hemodialysis with diabetes N=14**	***p*-value (ANOVA)**	***p*-value (2) vs (3)**
Age, y	46 (30-47)	53 (45-58)	49.5 (44-57)	0.164	0.529
Male (n, %)	13 (61.9)	15 (60)	11 (78.6)	0.473	0.304
Hs-CRP, mg/dl	0.11 (0.06-0.15)	0.63 (0.11-1.41)	0.97 (0.21-1.88)	<0.001	0.285
Lipid profile					
TC, mg/dl	181 (173-198)	182 (162-199)	175 (149-211)	0.863	0.803
TG, mg/dl	66 (49-98)	118 (95-207)	188 (141-242)	<0.001	0.076
LDL-c, mg/dl	99 (91-115)	96 (86-109)	94 (75-101)	0.153	0.110
HDL-c, mg/dl	56 (48-63)	42 (31-45.4)	38 (35-41)	<0.001	0.455
LDL subfractions					
L1%	88.6 (83.8-91.1)	84.1 (76.4-93.3)	74.1 (54.2-91.5)	0.056	0.092
L2%	5.0 (3.6-6.5)	8.5 (2.8-14.4)	16.1 (4.3-25.8)	0.022	0.092
L3%	4.1 (2.0-7.8)	4.9 (0.2-1.7)	7.2 (0.2-13.5)	0.491	0.260
L4%	1.0 (0.4-1.9)	0.6 (0.2-1.3)	1.6 (0.3-3.5)	0.208	0.164
L5%	0.4 (0.3-0.8)	1.9 (1.2-2.7)	2.2 (1.2-4.5)	<0.001	0.320
Endothelial function					
FMD, %	11.4 (9.0-15.1)	5.7 (4.4-6.8)	6.0 (4.3-7.3)	<0.001	0.953
